# Amygdalar reactivity is associated with prefrontal cortical thickness in a large population-based sample of adolescents

**DOI:** 10.1371/journal.pone.0216152

**Published:** 2019-05-02

**Authors:** Matthew D. Albaugh, James. J. Hudziak, Catherine Orr, Philip A. Spechler, Bader Chaarani, Scott Mackey, Claude Lepage, Vladimir Fonov, Pierre Rioux, Alan C. Evans, Tobias Banaschewski, Arun L. W. Bokde, Uli Bromberg, Christian Büchel, Erin Burke Quinlan, Sylvane Desrivières, Herta Flor, Antoine Grigis, Penny Gowland, Andreas Heinz, Bernd Ittermann, Jean-Luc Martinot, Marie-Laure Paillère Martinot, Frauke Nees, Dimitri Papadopoulos Orfanos, Tomáš Paus, Luise Poustka, Sabina Millenet, Juliane H. Fröhner, Michael N. Smolka, Henrik Walter, Robert Whelan, Gunter Schumann, Alexandra S. Potter, Hugh Garavan, IMAGEN Consortium

**Affiliations:** 1 Vermont Center for Children, Youth, and Families, Department of Psychiatry, University of Vermont College of Medicine, Burlington, VT, United States of America; 2 Department of Psychiatry, University of Vermont College of Medicine, Burlington, VT, United States of America; 3 McConnell Brain Imaging Centre, Montreal Neurological Institute, McGill University, Montreal, QC, Canada; 4 Department of Child and Adolescent Psychiatry and Psychotherapy, Central Institute of Mental Health, Medical Faculty Mannheim, Heidelberg University, Mannheim, Germany; 5 Discipline of Psychiatry, School of Medicine and Trinity College Institute of Neuroscience, Trinity College Dublin, Dublin, Ireland; 6 University Medical Centre Hamburg-Eppendorf, Hamburg, Germany; 7 Medical Research Council—Social, Genetic and Developmental Psychiatry Centre, Institute of Psychiatry, Psychology & Neuroscience, King’s College London, United Kingdom; 8 Department of Cognitive and Clinical Neuroscience, Central Institute of Mental Health, Medical Faculty Mannheim, Heidelberg University, Mannheim, Germany; 9 Department of Psychology, School of Social Sciences, University of Mannheim, Mannheim, Germany; 10 NeuroSpin, CEA, Université Paris-Saclay, Gif-sur-Yvette, France; 11 Sir Peter Mansfield Imaging Centre School of Physics and Astronomy, University of Nottingham, University Park, Nottingham, United Kingdom; 12 Charité –Universitätsmedizin Berlin, corporate member of Freie Universität Berlin, Humboldt-Universität zu Berlin, and Berlin Institute of Health, Department of Psychiatry and Psychotherapy, Campus Charité Mitte, Charitéplatz 1, Berlin, Germany; 13 Physikalisch-Technische Bundesanstalt (PTB), Braunschweig and Berlin, Germany [or depending on journal requirements can be: Physikalisch-Technische Bundesanstalt (PTB), Berlin, Germany; 14 Institut National de la Santé et de la Recherche Médicale, INSERM Unit 1000 “Neuroimaging & Psychiatry”, University Paris Sud, University Paris Descartes—Sorbonne Paris Cité; and Maison de Solenn, Paris, France; 15 Institut National de la Santé et de la Recherche Médicale, INSERM Unit 1000 “Neuroimaging & Psychiatry”; University Paris Sud; University Paris Descartes; Sorbonne Universités; and AP-HP, Department of Child and AdolescentPsychiatryPitié-Salpêtrière Hospital, Paris, France; 16 Bloorview Research Institute, Holland Bloorview Kids Rehabilitation Hospital and Departments of Psychology and Psychiatry, University of Toronto, Toronto, Ontario, Canada; 17 Department of Child and Adolescent Psychiatry and Psychotherapy, University Medical Centre Göttingen, Göttingen, Germany; 18 Department of Psychiatry and Neuroimaging Center, Technische Universität Dresden, Dresden, Germany; 19 School of Psychology and Global Brain Health Institute, Trinity College Dublin, Ireland; RWTH Aachen, GERMANY

## Abstract

In structural neuroimaging studies, reduced cerebral cortical thickness in orbital and ventromedial prefrontal regions is frequently interpreted as reflecting an impaired ability to downregulate neuronal activity in the amygdalae. Unfortunately, little research has been conducted in order to test this conjecture. We examine the extent to which amygdalar reactivity is associated with cortical thickness in a population-based sample of adolescents. Data were obtained from the IMAGEN study, which includes 2,223 adolescents. While undergoing functional neuroimaging, participants passively viewed video clips of a face that started from a neutral expression and progressively turned angry, or, instead, turned to a second neutral expression. Left and right amygdala ROIs were used to extract mean BOLD signal change for the angry minus neutral face contrast for all subjects. T1-weighted images were processed through the CIVET pipeline (version 2.1.0). In variable-centered analyses, local cortical thickness was regressed against amygdalar reactivity using first and second-order linear models. In a follow-up person-centered analysis, we defined a “high reactive” group of participants based on mean amygdalar BOLD signal change for the angry minus neutral face contrast. Between-group differences in cortical thickness were examined (“high reactive” versus all other participants). A significant association was revealed between the continuous measure of amygdalar reactivity and bilateral ventromedial prefrontal cortical thickness in a second-order linear model (*p* < 0.05, corrected). The “high reactive” group, in comparison to all other participants, possessed reduced cortical thickness in bilateral orbital and ventromedial prefrontal cortices, bilateral anterior temporal cortices, left caudal middle temporal gyrus, and the left inferior and middle frontal gyri (*p* < 0.05, corrected). Results are consistent with non-human primate studies, and provide empirical support for an association between reduced prefrontal cortical thickness and amygdalar reactivity. Future research will likely benefit from investigating the degree to which psychopathology qualifies relations between prefrontal cortical structure and amygdalar reactivity.

## Introduction

Among primates, dense anatomical connections exist between regions of the prefrontal cortex and the amygdalae [1–4]. Given these anatomical connections, it has long been posited that prefrontal regions provide “top-down” modulation of amygdalar functioning [1–4]. In support of this notion, functional magnetic resonance imaging (fMRI) studies of emotion regulation have implicated prefrontal regions in the regulation of amygdalar activity [5–12]. Specifically, across such studies, effective forms of emotion regulation have been associated with increased activation in prefrontal areas, as well as concomitant decreases in amygdalar activation.

In surface-based studies of human cortical morphology, reduced cortical thickness in prefrontal areas—particularly in orbital and ventromedial prefrontal regions—has commonly been interpreted as reflecting an impaired ability to regulate limbic structures like the amygdalae [13–15]. Despite the prevalence of such conjecture, little research has been performed in order to directly test this speculation. To our knowledge, only one study has directly tested the extent to which cerebral cortical thickness is associated with amygdalar reactivity [16]. Studying 20 healthy human adults (12 males, 8 females; mean age, 35.1 ± 12.7 years), Foland-Ross et al. (2010) tested the extent to which activation in the left amygdala during cognitive evaluation of negative emotional facial expressions was related to prefrontal cortical thickness. Specifically, during cognitive evaluation of negative emotional facial expressions, participants chose one of two words that best described the emotional face presented on the screen. Citing a host of prior animal and human studies demonstrating suppression of amygdalar activity by ventral prefrontal cortical areas [14, 17–21], the authors hypothesized that participants with reduced prefrontal cortical thickness would exhibit greater amygdalar activation—reflecting a diminished capacity to downregulate the amygdalae during the affect labeling task. As hypothesized, the authors found that amygdalar activation during the labeling task was negatively correlated with cortical thickness in the left ventromedial prefrontal cortex (vmPFC).

It remains unclear if such structure-function relations exist across the developmental span, including during childhood and adolescence. Given that adolescence is accompanied by a dramatic increase in mood disorders [22], characterizing fronto-limbic relations during this developmental window may help shed light on neurodevelopmental processes associated with the emergence of psychopathology. Further, it is possible that sex qualifies relations between cortical structure and amygdalar reactivity; unfortunately, prior research may not have been adequately powered to detect sex differences. Indeed, a growing literature indicates that sex hormone levels in developing youths influence cortico-limbic maturation, including fronto-amygdalar networks [23–27]. Similarly, recent resting state functional connectivity research suggests unique patterns of cortico-amygdalar connectivity between sexes during adolescence [28].

In the present study, we investigate the extent to which amygdalar reactivity to angry faces is associated with cerebral cortical thickness in a large population-based sample of adolescents. Based on non-human primate tracer studies of fronto-amygdalar anatomical connectivity, we hypothesize that reduced cortical thickness in ventromedial prefrontal cortices will be associated with increased amygdalar activation to negatively valenced emotional stimuli. We utilize a publicly available probabilistic atlas of vmPFC cytoarchitecture in an attempt to reveal which vmPFC subdivisions are most significantly associated with amygdalar reactivity (S1 Fig). We also investigate the degree to which sex qualifies the relationship between cerebral cortical structure and amygdalar reactivity.

## Materials and methods

### Participants

Neuroimaging and behavioral data were obtained from the IMAGEN study conducted across 8 European sites in France, the United Kingdom, Ireland, and Germany, which includes 2,223 adolescents recruited from schools at age 14 years. Local ethics research committees approved the study at each site (London: Psychiatry, Nursing and Midwifery (PNM) Research Ethics Subcommittee (RESC), Waterloo Campus, King's College London. Nottingham: University of Nottingham Medical School Ethics Committee. Mannheim: Medizinische Fakultaet Mannheim, Ruprecht Karl Universitaet Heidelberg and Ethik-Kommission II an der Fakultaet fuer Kliniksche Medizin Mannheim. Dresden: Ethikkommission der Medizinischen Fakultaet Carl Gustav Carus, TU Dresden Medizinische Fakultaet. Hamburg: Ethics board, Hamburg Chamber of Physicians. Paris: CPP IDF VII (Comité de protection des personnes Ile de France), ID RCB: 2007-A00778-45 September 24th 2007. Dublin: TCD School of Psychology REC. Berlin: ethics committee of the Faculty of Psychology). Written consent was obtained from the parent or guardian as well as verbal assent from the adolescent. A detailed description of recruitment and assessment procedures has been published elsewhere [29]. In the present study, a total of 1,753 participants possessed quality controlled neuroimaging data and complete demographic data.

### Demographic measures

The puberty development scale (PDS) was administered to assess the pubertal status of study participants [30]. The socioeconomic status (SES) score was derived by summing the following variables: Mother’s Education Score, Father’s Education Score, Family Stress Unemployment Score, Financial Difficulties Score, Home Inadequacy Score, Neighborhood Score, Financial Crisis Score, Mother Employed Score, and Father Employed Score [31].

### MRI acquisition

MRI scanning was conducted at the eight IMAGEN assessment sites using 3T whole body MRI systems [29]. Participants underwent MRI scanning for approximately one hour in order to collect a combination of structural and functional imaging data. 3D T1-weighted images were acquired using a magnetization prepared gradient echo sequence based on the ADNI protocol (http://adni.loni.usc.edu/methods/mri-tool/mri-analysis/), which utilizes protocols developed to minimize image differences across scanner makes and models. With regard to the functional task used in the present study, 160 volumes were obtained per participant, with each volume consisting of 40 slices. Slices were aligned relative to the anterior commissure—posterior commissure line (2.4 mm thickness, 1 mm gap, TR = 2.20 s, TE = 30 ms). Please see Schumann et al. (2010) for further details.

### Processing of functional MRI

In the faces fMRI task, participants passively viewed video clips that contained either a person's face or a control stimulus. This task was created by Grosbras and Paus (2006) and required participants to passively view a series of short (2–5 s) video clips displaying a face that started from a neutral expression and progressively turned angry, or, progressively turned to a second neutral expression [32]. The control stimuli contained expanding and contracting concentric circles of various contrasts, roughly matching the contrast and motion characteristics of the face stimuli. These control images were created and originally implemented by Beauchamp et al. (2003) and were included to account for neural activity associated with viewing non-biological motion [33]. All stimuli were presented as 18 s blocks, with 4–7 video clips per block during a face block. Each run was comprised of 5 blocks of neutral faces and 5 blocks of angry faces.

Pre-processing of echo-planar imaging data was performed using SPM8 (Statistical Parametric Mapping, http://www.fil.ion.ucl.ac.uk/spm/). Time series data were initially corrected for slice timing, and subsequently corrected for movement, non-linearly warped into MNI space, and spatially smoothed at 5 mm-FWHM. Functional activation maps were generated with SPM8 and regressed using a general linear model with AR noise model against a design-matrix modeling each 18 second block of stimulus presentation. Contrast images were obtained for the main effect of angry faces and neutral faces, as well as for differential activation of angry minus neutral faces. Left and right amygdala ROIs (from the Harvard-Oxford Subcortical Atlas, thresholded at 50 percent probability and binarized) were used to extract mean BOLD signal change for the angry face minus neutral face contrast for all subjects (S2 Fig).

### Processing of structural MRI

Quality controlled native MR images were processed through the CIVET pipeline (version 2.1.0) using the CBRAIN platform [34]. As described in detail previously [35], the following steps were performed as part of the CIVET pipeline [36, 37]. First, native MR images were linearly registered to a standardized MNI-Talairach space based on the ICBM152 dataset in order to account for volumetric differences between subjects [38–40]. Second, the N3 algorithm was implemented in order to correct for intensity non-uniformity artifacts [41]. Third, classification of white matter (WM), gray matter (GM), and cerebrospinal fluid (CSF) was performed using the INSECT algorithm [42]. Fourth, the CLASP algorithm was used to generate high-resolution hemispheric surfaces (40,962 vertices per hemisphere) [43–46]. Hemispheric surfaces were generated for both the WM/GM interface and GM/CSF interface. Fifth, surfaces for each hemisphere were non-linearly registered to an average surface created from the ICBM152 dataset [39, 44, 47]. A reverse linear transformation was carried out on each subject’s images, and cortical thickness estimations were calculated at each cortical point in native space using the *t*link metric [48, 49]. As a final step, subjects’ cortical thickness maps were blurred using a 20-millimeter full width at half maximum surface-based diffusion smoothing kernel [50], providing optimal sensitivity for cortical thickness analysis [49].

### Statistical analysis

Cortical thickness analyses were performed using SurfStat, a toolbox created for MATLAB (The MathWorks, Inc., Natick, Massachusetts) by Dr. Keith Worsley (http://www.math.mcgill.ca/keith/surfstat/). First, local cerebral cortical thickness was regressed against the continuous measure of amygdalar reactivity (i.e., mean amygdalar BOLD signal change for angry minus neutral face contrast) using first and second-order linear models:

Y = 1 + b_1_Amy + b_2_Age + b_3_Sex + b_4_Site + b_5_Hand + b_6_TBV + b_7_PDS + b_8_SES + b_9_IQPR + b_10_IQVC

*Y = 1 + b*_*1*_*Amy + b*_*2*_*Amy^2 + b*_*3*_*Age + b*_*4*_*Sex + b*_*5*_*Site + b*_*6*_*Hand + b*_*7*_*TBV + b*_*8*_*PDS + b*_*9*_*SES + b*_*10*_*IQPR + b*_*11*_*IQVC*.

where “Amy” refers to the angry minus neutral face contrast value. In a follow-up person-centered analysis, we defined a “high reactive” group as participants falling 1.5 standard deviations above mean amygdalar BOLD signal change for the angry minus neutral face contrast (corresponding, approximately, to the upper 5% of participants in the present sample). Imposing this statistical cut-off resulted in 90 “high reactive” participants, and 1663 controls. Between-group differences in cortical thickness were examined (“high reactive” versus all other participants) using the following model:

Y = 1 + b_1_Group + b_2_Age + b_3_Sex + b_4_Site + b_5_Hand + b_6_TBV + b_7_PDS + b_8_SES + b_9_IQPR + b_10_IQVC

Age, total brain volume, sex, site, handedness, SES, Performance IQ, Verbal IQ, and pubertal development were controlled for in all vertex-wise surface-based analyses (both variable- and person-centered analyses). In order to examine the extent to which the association between amygdalar reactivity and cortical thickness was qualified by sex, a “sex by Amy” interaction term was tested in first and second-order linear models. Similarly, a “sex by Group” interaction term was added to the model used in the follow-up analysis.

To account for multiple comparisons, random field theory (RFT) correction was applied to the entire cortical surface [51]. In order to identify significant clusters, an initial height threshold of p ≤ .001 was implemented at the vertex level, and a corrected familywise error (p ≤ .05) was subsequently applied. Further, vertex-level RFT thresholding was implemented using the vertex-wise RFT critical *t*-value which was calculated from the expected Euler characteristic and number of resolution elements, or “resels” [51].

## Results

### Demographic measures

Demographic information for participants is summarized in Table 1. Participants in the “high reactive” group possessed significantly lower Performance IQs relative to all other participants. No other significant differences were revealed between groups.

**Table 1:** Demographic summary. Demographic summary for amygdalar reactivity groups.

	High Reactive Group *M(SD)* (*n* = 90)	Control *M(SD)* (*n* = 1663)	*t* or *X*^*2*^ value	*p* value
**Age (yrs)**	14.42(0.42)	14.43(0.41)	0.111	0.912
**Sex**	Males = 51(56.7%)	Males = 797(47.9%)	2.612	0.106
**SES**	17.43(3.71)	17.90(3.92)	1.108	0.268
**IQPR**	103.77(13.81)	108.21(14.06)	**2.918**	0.004*
**IQVC**	109.62(16.04)	110.54(14.03)	0.602	0.547
**PDS**	2.87(0.55)	2.91(0.56)	0.724	0.469
**Brain Volume (mm**^**3**^**)**	1442.20(134.40)	1425.22(131.59)	-1.191	0.234

SES = socioeconomic status; puberty = pubertal development scale; IQ PR = performance IQ; IQ VC = verbal IQ

* = *p* < 0.007 (corrected significance value)

### Amygdalar reactivity and cortical thickness

**Fig 1:**
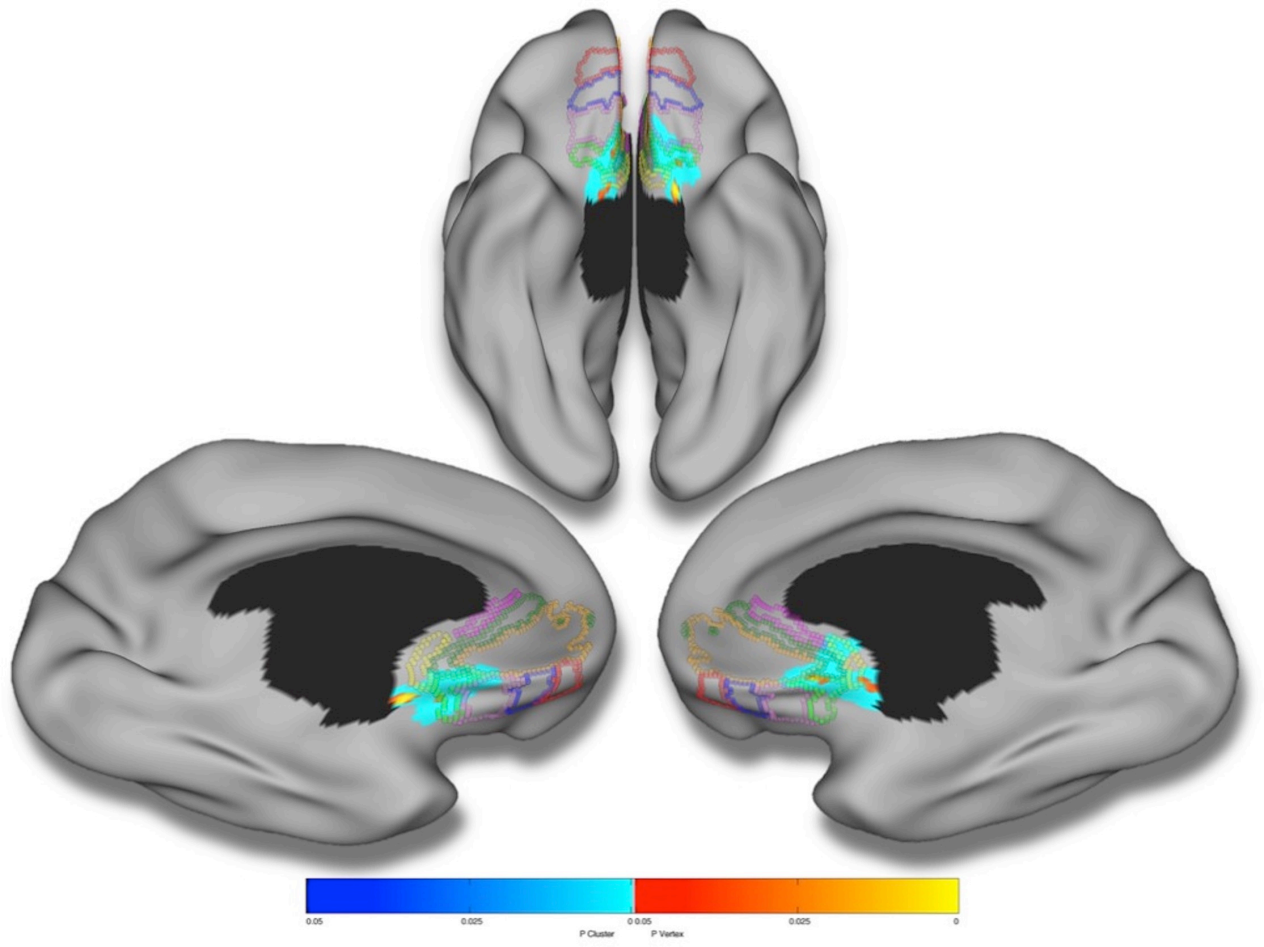
Amygdalar reactivity and cortical thickness. Brain areas in which local cerebral cortical thickness is associated with the continuous measure of amygdalar activation (i.e., angry minus neutral face contrast) in a second-order (quadratic) model over the whole sample (n = 1753). Figure is shown at *p* ≤ 0.05, RFT corrected. Blue areas are significant at the cluster level and red color corresponds to areas significant at the vertex level. Controlled for age, total brain volume, sex, site, handedness, Performance IQ, Verbal IQ, SES and pubertal development. Colored borders correspond to the maximum symmetric probability map derived from the cytoarchtectonic studies of Mackey & Petrides (2014).

No significant first-order linear associations were found between the continuous measure of amygdalar reactivity and cerebral cortical thickness. Testing of a second-order linear model revealed a significant quadratic association between amygdalar reactivity and ventromedial prefrontal cortical thickness (Fig 1 and Table 2). Applying the Mackey and Petrides (2014) human vmPFC atlas, significant cluster-wise associations were revealed, bilaterally, in areas 25, 14c, 14m, 14r, and 32 [52].

**Table 2:** Peak areas from variable-centered analysis. Peak areas in which local cerebral cortical thickness was associated with the continuous measure of amygdalar activation (i.e., angry minus neutral face contrast) in a second-order (quadratic) model over the whole sample.

Peak Vertex Location	t-statistic	MNI Coordinates
**Left frontal orbital cortex**	-4.43	-15.82, -1.38, -13.56
**Right subcallosal cortex**	-4.15	4.73, 16.55, -22.88
**Right subcallosal and frontal orbital cortex**	-4.11	7.51, 6.91, -14.99
**Left subcallosal and frontal orbital cortex**	-4.08	-14.81, 10.24, -17.28

**Fig 2:**
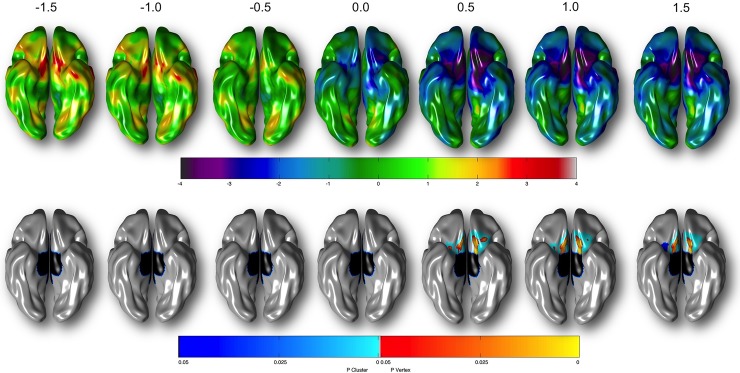
Association between amygdalar reactivity and cortical thickness at varying contrast levels. Relationship between cortical thickness and angry minus neutral face contrast value (averaged across bilateral amygdalar ROI) at varying levels of angry minus neutral face contrast values (-1.5, -1.0, -0.5, 0.0, 0.5, 1.0, 1.5). In top row, colors represent t-statistic values associated with regression coefficient. Bottom row depicts RFT-corrected results (*p* ≤ 0.05). Blue areas are significant at the cluster level and red color corresponds to areas significant at the vertex level. Controlled for age, total brain volume, sex, site, handedness, Performance IQ, Verbal IQ, SES and pubertal development.

Probing of the quadratic association revealed a weak non-significant positive association between cortical thickness and amygdalar reactivity at negative values for the angry minus neutral face contrast; however, this pattern reversed such that a significant inverse association between cortical thickness and amygdalar reactivity was observed at positive (≥0.5) angry minus neutral face contrast values (Fig 2 and S3 Fig).

**Fig 3:**
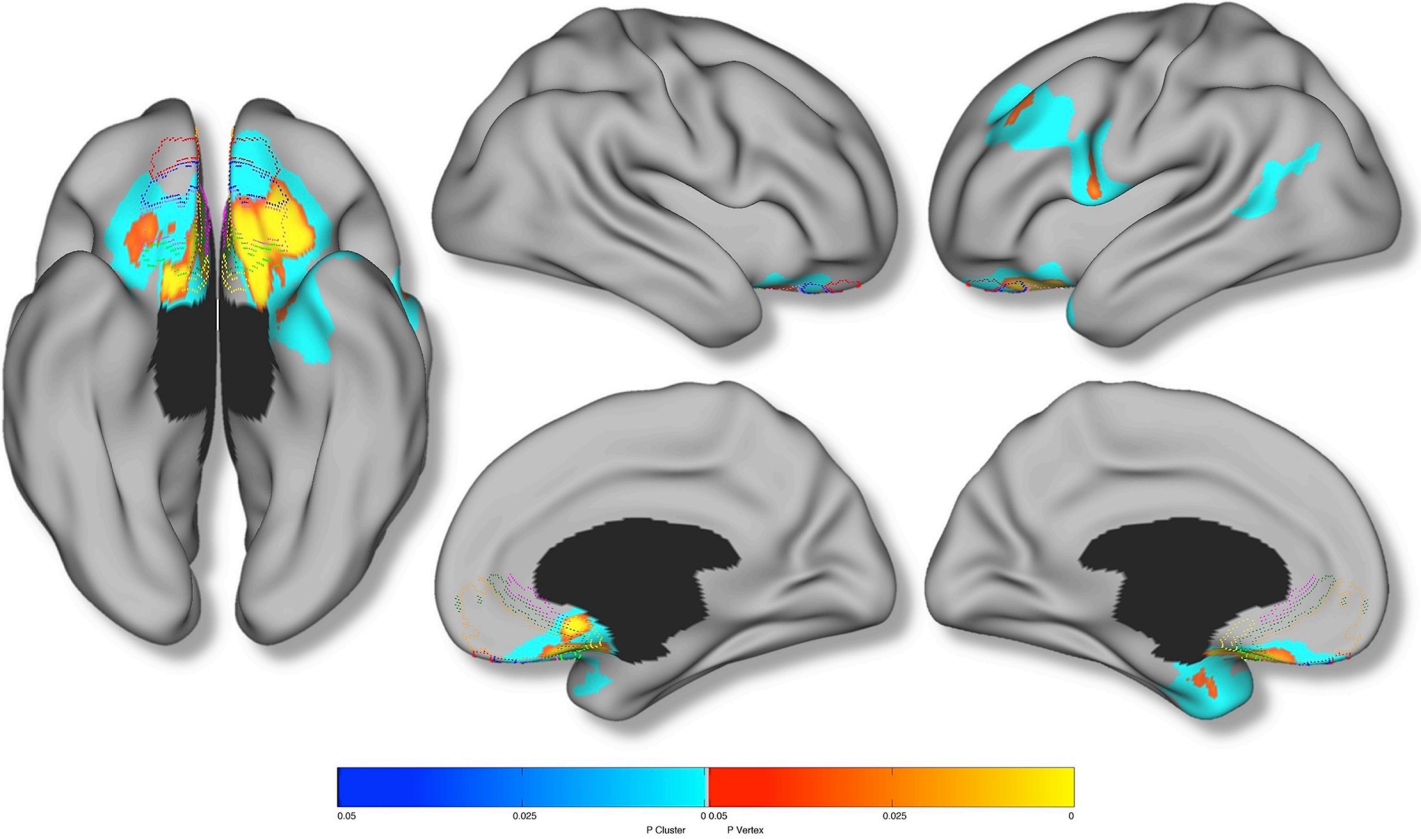
Results of “high reactive” group analysis. Brain areas in which local cerebral cortical thickness was significantly reduced in the “high reactive” group (n = 90) relative to all other participants (n = 1663). Random field theory was used to correct for multiple comparisons over the entire cortical mantle. Figure is shown at *p* ≤ 0.05, RFT corrected. Blue areas are significant at the cluster level and red color corresponds to areas significant at the vertex level. Controlled for age, total brain volume, sex, site, handedness, Performance IQ, Verbal IQ, SES and pubertal development. Colored borders correspond to the maximum symmetric probability map derived from the cytoarchtectonic studies of Mackey & Petrides (2014).

Group analyses revealed that the “high reactive” group, in comparison to all other participants, possessed reduced cortical thickness in bilateral orbital and ventromedial prefrontal cortices, bilateral anterior temporal cortices, left caudal middle temporal gyrus, as well as portions of the left inferior and middle frontal gyri (*p* < 0.05, RFT corrected) (Fig 3 and Table 3). Given the difference in group sizes, we subsequently conducted a Levene’s test in order to test for potential heteroscedasticity. Importantly, in all significant cortical regions, there was no evidence of heteroscedasticity. Applying the Mackey and Petrides (2014) human vmPFC atlas, significant cluster-wise associations were revealed in all cytoarchitectonic subdivisions with the exception of right area 11m and left area 24 [52].

**Table 3:** Peak areas from person-centered analysis. Peak areas in which local cerebral cortical thickness was significantly reduced in the “high reactive” group relative to all other participants.

Peak Vertex Location	t-statistic	MNI Coordinates (x,y,z)
**Left subcallosal and frontal orbital cortex**	-5.23	-11.49, 14.36, -15.67
**Right subcallosal cortex**	-4.87	3.87, 16.10, -7.56
**Left precentral and inferior frontal gyrus**	-4.19	-53.88, 6.97, 8.79
**Left middle temporal gyrus and angular gyrus**	-3.83	-63.43, -48.02, 6.16

In both variable- and person-centered analyses, the relationship between amygdalar reactivity and cerebral cortical thickness was not moderated by sex.

## Discussion

In many structural neuroimaging studies, reduced cortical thickness in orbitofrontal and ventromedial prefrontal areas has been interpreted as reflecting an impaired ability to downregulate amygdalar regions. To our knowledge, this is the largest multimodal neuroimaging study to provide support for this widespread speculation. Specifically, using both variable- and person-centered approaches, we revealed an association between high amygdalar reactivity to emotional stimuli and reduced ventromedial prefrontal cortical thickness in a large, population-based sample of adolescents. Further, results from the present study suggest that the relationship between cerebral cortical thickness and amygdalar reactivity is not influenced by sex.

As hypothesized, a continuous measure of amygdalar reactivity to angry faces was associated with cortical thickness in the vmPFC, including portions of the right subgenual anterior cingulate—areas known to have strong anatomical connections with the amygdalae [53]. In particular, analyzing the entire population-based sample of adolescents, we found evidence of a significant quadratic association between amygdalar reactivity and bilateral ventromedial prefrontal cortical thickness. Post hoc probing of this curvilinear relationship revealed significant inverse associations between amygdalar reactivity and ventromedial prefrontal cortical thickness at moderate to high (≥0.5) angry minus neutral face contrast values. These findings appear consistent with the notion of thinner ventromedial prefrontal cortices being tied to a diminished capacity to regulate amygdalar activation in response to negatively valenced emotional stimuli [16].

Using a person-centered approach, we found that the “high reactive” group, in comparison to all remaining participants, possessed reduced cortical thickness in bilateral orbital and ventromedial prefrontal cortices, bilateral anterior temporal cortices, left caudal middle temporal gyrus, and portions of the left dorsolateral prefrontal cortex. These results are consistent with non-human primate tracer studies indicating that caudal orbital and medial prefrontal cortices possess the densest anatomical connections with amygdalar regions. Present findings are also consistent with the only prior study to investigate the relationship between cortical thickness and amygdalar reactivity [16]. As noted, however, this prior study was conducted using a relatively small number of healthy adult participants.

Participants in the “high reactive” group exhibited reduced cerebral cortical thickness in dorsolateral prefrontal regions. To our knowledge, this is the first study to demonstrate an association between cortical thickness in dorsolateral prefrontal areas and amygdalar reactivity. This result is somewhat surprising given that dorsolateral prefrontal areas do not possess strong anatomical connections with the amygdalae [1, 2, 4]. Nonetheless, the dorsolateral prefrontal cortex has long been implicated in cognitive, or voluntary, aspects of emotion regulation. In functional neuroimaging studies, voluntary forms of emotion regulation (e.g., cognitive reappraisal) have been consistently tied to increased activation in dorsolateral prefrontal regions, and concomitant decreases in amygdalar activity [5–10]. As others have previously suggested, it is likely that dorsal prefrontal regions influence amygdalar activity through phylogenetically older areas of the cerebral cortex—such as the vmPFC—that possess anatomical connections with the amygdalae [17]. Dovetailing with functional imaging studies of cognitive emotion regulation, resting state functional connectivity studies of the amygdalae indicate that amygdalar activity, at rest, is negatively associated with activity in dorsal prefrontal and inferior parietal cortices [54–56]. Results from the present study provide further support for functional antagonism between portions of the DLPFC and the amygdalae.

Whereas Foland-Ross (2010) examined the relationship between amygdalar activation and ventromedial prefrontal cortical thickness during cognitive evaluation of negative emotional faces (i.e., affect labeling task), the present study utilized a functional probe that involved only passive viewing of neutral and negative emotional faces. Thus, our results suggest that amygdalar reactivity is related to ventromedial prefrontal cortical thickness during passive viewing of emotional stimuli, and further support the vmPFC’s putative role in automatic or involuntary aspects of human emotion regulation [57].

Several limitations of the present study should be noted. In rodent models of chronic stress, structural changes and increased neuronal excitability have been reported in the amygdalae [58–60]. Furthermore, there is evidence that such functional and structural changes in the amygdalae undergird the emergence of anxiety-like symptoms in rodent models of chronic stress [58–60]. That being said, we can only speculate as to the developmental origins of the observed structure-function relationship in the present study. As members of our group have previously discussed [35], it is possible that reduced thickness in prefrontal regulatory regions—reflecting compromised cytoarchitectonic integrity—results in a diminished capacity to downregulate amygdalar activity. It is also possible that increased amygdalar reactivity, over time, results in structural damage to prefrontal cortices through continued activation of the hypothalamic-pituitary-adrenal (HPA) axis and resultant release of cortisol [61–64]. Both of these processes could potentially account for the structure-function association in the present study; future studies, however, are needed to directly test these potential explanations. We cannot rule out the possibility that structure-function relations observed in the present study reflect parallel, experience-driven developmental processes that are independent of underlying anatomical connectivity. With regard to our group analyses, it should be noted that the “high reactive” group possessed significantly lower Performance IQs relative to all other participants. We cannot rule out the possibility that this difference in Performance IQ may have contributed to the observed cortical thickness differences. To address this issue, we examined the relationship between Performance IQ and cortical thickness while controlling for age, total brain volume, sex, site, handedness, SES, Verbal IQ, and pubertal development. Critically, no significant associations were revealed between Performance IQ and cortical thickness. Further, no trend-level associations (p<0.005 uncorrected) were observed in any of the cortical regions that were related to amygdalar reactivity (in both whole sample second-order linear model results, and group results). Given the age of participants in the present study, it is unclear the extent to which our findings generalize to adult populations. Importantly, the cerebral cortex and limbic structures are still undergoing significant structural change during this developmental period [65–67], and evidence from prior imaging studies indicates that white matter pathways serving to connect the amygdalae and prefrontal cortices are continuing to mature during adolescence [68–70]. Given dynamic changes in brain structure and connectivity during adolescence, caution should be taken in extending the present findings to children and adults. Lastly, in our variable-centered analysis, it should be noted that the effect size of the observed quadratic association was relatively small (*R*^*2*^ = 0.013). Ventromedial prefrontal cortical thickness is likely just one of myriad brain factors associated with amygdalar reactivity. Factors such as white matter microstructure in pathways such as the uncinate fasciculus may be important moderating factors when assessing the association between cortical structure and amygdalar reactivity. Future multimodal studies are needed to more fully elucidate such relations.

It is noteworthy that results of the present study appear consistent with a prior report of structural covariance between amygdalar volume and cerebral cortical thickness in a large sample of typically developing youths [35]. In particular, Albaugh et al. (2013) found that amygdalar volume was negatively associated with cortical thickness in orbitofrontal, ventromedial, and dorsolateral prefrontal cortices. A similar pattern of results has been reported in a large adult sample [71].

Although the aim of the present study was to characterize relations between amygdalar reactivity and cerebral cortical structure, future studies will likely benefit from investigating the extent to which forms of psychopathology moderate these structure-function relations. It is possible that such relations between cerebral cortical structure and amygdalar reactivity may not only have ties to concomitant psychopathology, but also have predictive utility for the emergence of future psychopathology. In addition, ongoing prospective longitudinal studies, such as the Adolescent Brain and Cognitive Development (ABCD) study, may help to shed light on how the observed relations between cerebral cortical structure and amygdalar reactivity develop across childhood and adolescence.

## Supporting information

S1 Fig Cytoarchitectonic-based atlas. Surface-based representation of maximum symmetric probability map derived from the cytoarchitectonic studies of Mackey & Petrides (2014). Colors correspond to the following cytoarchitectonic areas: red = 11m; blue = 14r’; pink lavender = 14r; lime green = 14c; yellow = 25; orange = 14m; dark green = 32; magenta = 24.
(TIF)

S2 Fig Amygdala ROIs. Depiction of left and right amygdala ROIs (from the Harvard-Oxford Subcortical Atlas, thresholded at 50 percent probability and binarized) that were used to extract the mean BOLD signal from the angry face minus neutral face contrast.
(TIF)

S3 Fig Scatter plot of quadratic association. Scatter plot depicting quadratic association between residualized average thickness of right vmPFC cluster (adjusted for age, total brain volume, sex, site, handedness, Performance IQ, Verbal IQ, SES and pubertal development) and angry minus neutral face contrast value (mean value for left and right amygdalae).
(TIF)
