# Supplementary figures and images for: Amygdalar reactivity is associated with prefrontal cortical thickness in a large population-based sample of adolescents
Source: PLoS One. 2019 May 2;14(5):e0216152. doi: 10.1371/journal.pone.0216152 (PMC6497259; doi:10.1371/journal.pone.0216152)

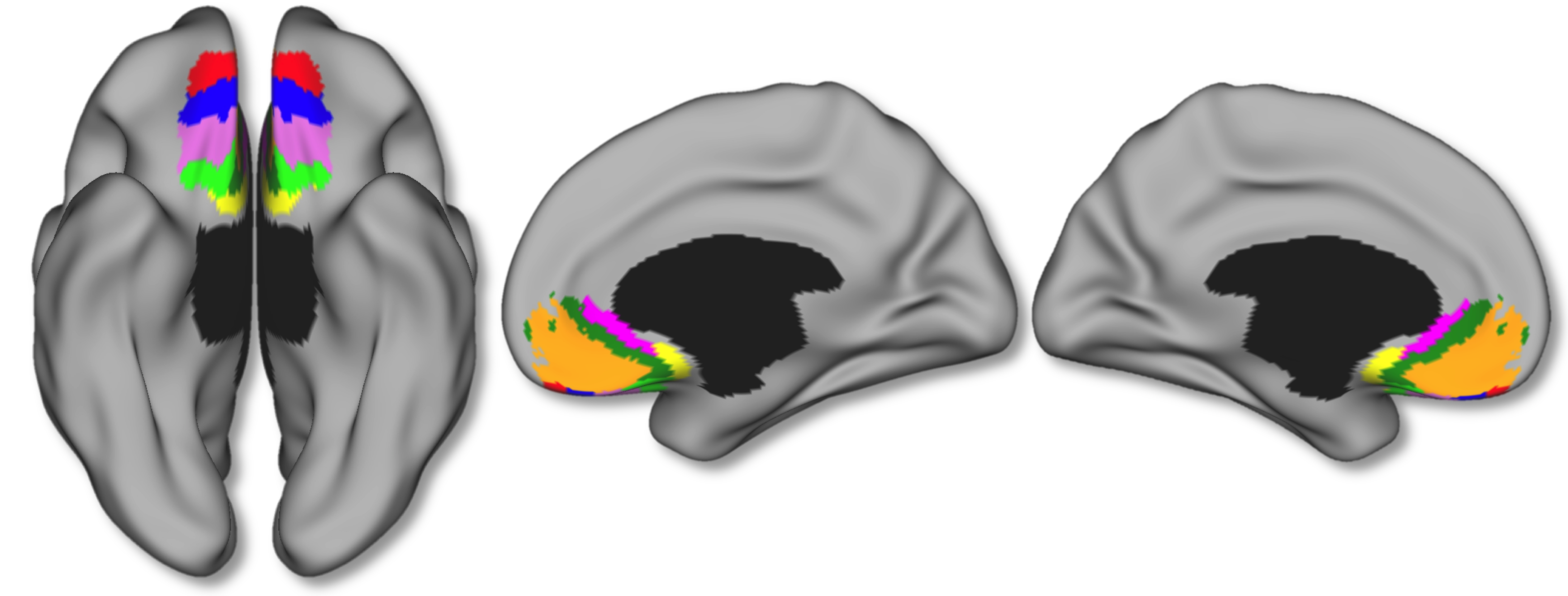

Supplement: S1 Fig — Surface-based representation of maximum symmetric probability map derived from the cytoarchitectonic studies of Mackey & Petrides (2014). Colors correspond to the following cytoarchitectonic areas: red = 11m; blue = 14r’; pink lavender = 14r; lime green = 14c; yellow = 25; orange = 14m; dark green = 32; magenta = 24. (TIF) [file pone.0216152.s001.tif]

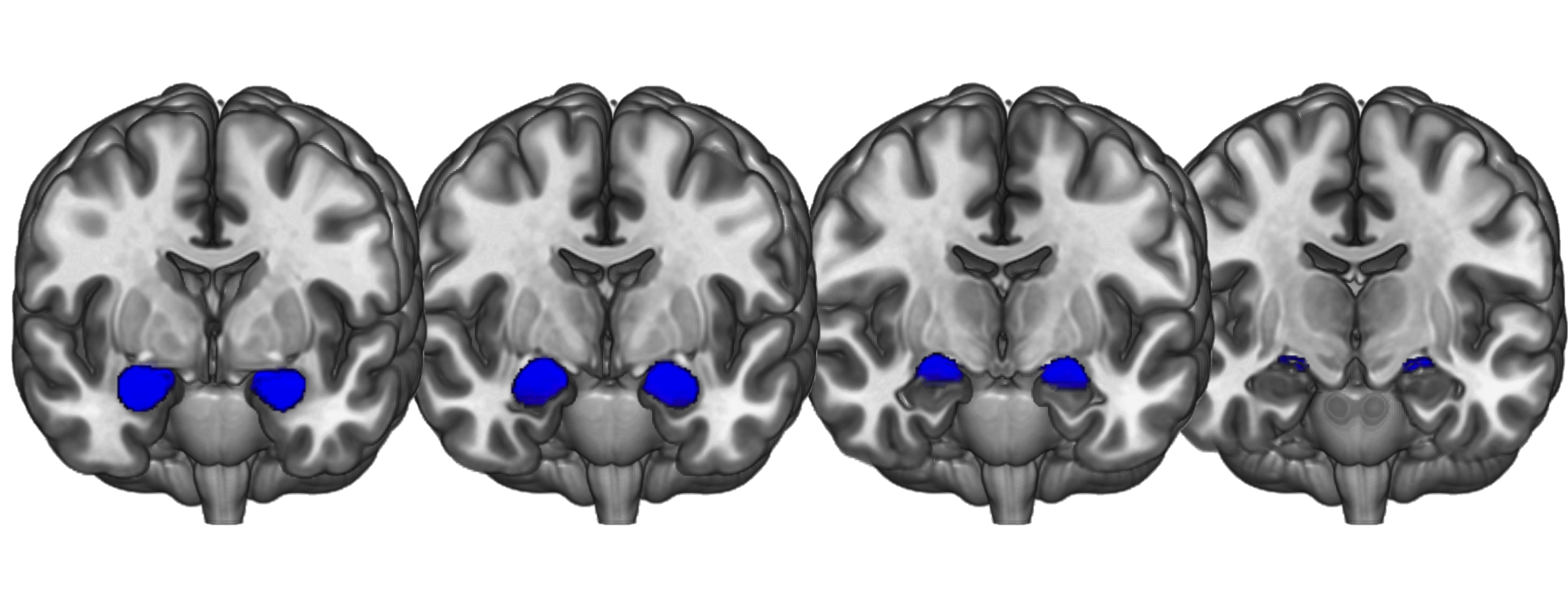

Supplement: S2 Fig — Depiction of left and right amygdala ROIs (from the Harvard-Oxford Subcortical Atlas, thresholded at 50 percent probability and binarized) that were used to extract the mean BOLD signal from the angry face minus neutral face contrast. (TIF) [file pone.0216152.s002.tif]

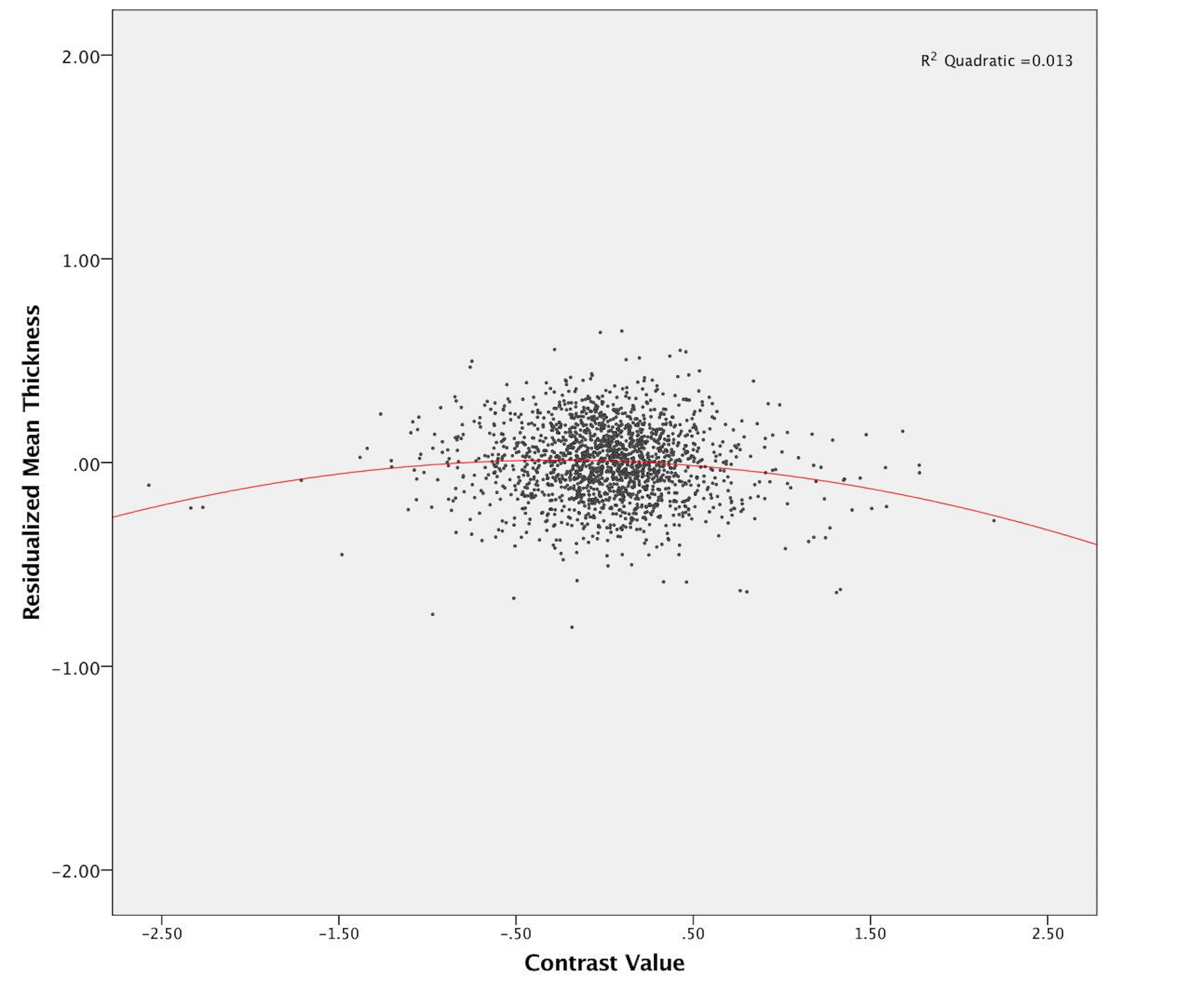

Supplement: S3 Fig — Scatter plot depicting quadratic association between residualized average thickness of right vmPFC cluster (adjusted for age, total brain volume, sex, site, handedness, Performance IQ, Verbal IQ, SES and pubertal development) and angry minus neutral face contrast value (mean value for left and right amygdalae). (TIF) [file pone.0216152.s003.tif]
